# A rare case of large pyosalpinx in an elderly patient with well-controlled type 2 diabetes mellitus: a case report

**DOI:** 10.1186/s13256-018-1841-6

**Published:** 2018-10-06

**Authors:** Mayuko Hida, Takatoshi Anno, Fumiko Kawasaki, Hideaki Kaneto, Kohei Kaku, Niro Okimoto

**Affiliations:** 1 Department of General Internal Medicine 1, Kawasaki Medical School, 2-6-1 Nakasange, Kita-ku, Okayama, 700-8505 Japan; 2 Department of Diabetes, Metabolism and Endocrinology, Kawasaki Medical School, Kurashiki, 701-0192 Japan

**Keywords:** Case report, Pyosalpinx, Type 2 diabetes mellitus, Elderly patient

## Abstract

**Background:**

Pyosalpinx, which is one of the pelvic inflammatory diseases, is usually observed in young women; it is rarely found in older women. Possible causative agents are thought to be *Chlamydia trachomatis* and *Neisseria gonorrhea* in addition to some *Enterobacteriaceae*. On the other hand, type 2 diabetes is a disease with a lot of complications such as hyperglycemia, inflammation, and immune disorders. Therefore, patients with type 2 diabetes mellitus have an increased susceptibility to infection especially when glycemic control is poor.

**Case presentation:**

We experienced a rare case of large pyosalpinx in an elderly patient with well-controlled type 2 diabetes mellitus. A 72-year-old Japanese woman with a 10-year history of type 2 diabetes mellitus had symptoms of diarrhea and persistent pain in left lower abdomen. She had mild tenderness to palpation in her abdomen. Inflammation markers were markedly elevated. Her abdominal computed tomography and magnetic resonance imaging on admission revealed a tumor in left side of intrapelvis and we diagnosed her as having pyosalpinx. Pathogenic bacteria were not detected. On admission, her glycemic control was relatively good; in addition, her glycated hemoglobin levels were around 6% for over 1 year. Although pathogenic bacteria were not detected, we started antibiotics therapy. Fourteen days after starting the antibiotics her laboratory data were improved. Three months later, the tumor was markedly smaller compared to that on admission.

**Conclusions:**

We should keep in mind that older patients with type 2 diabetes mellitus are immunocompromised hosts and thereby they could have rare pelvic inflammatory disease such as pyosalpinx even when good glycemic control is obtained for a long period of time.

## Background

Pyosalpinx, which is one of the pelvic inflammatory diseases (PIDs), is the collection of pus in the fallopian tubes and it is a sexually transmitted infection in many cases. Therefore, pyosalpinx is usually observed in young women; it is rarely found in older women [1]. In fact, it is thought that youth, menstruation, tobacco smoking, and having many sexual partners are the risk factors for pyosalpinx [2] and that the presence of pyosalpinx leads to infertility and/or the development of iliopsoas abscess. Possible causative agents are thought to be *Chlamydia trachomatis* and *Neisseria gonorrhea* in addition to some *Enterobacteriaceae*. In many cases, however, causative bacteria are not necessarily detected. Moreover, the clinical presentation of PID is often nonspecific and the correct diagnosis may be difficult at first based on the results of imaging studies [3]. Although it is rare for physicians to select magnetic resonance imaging (MRI) as a first choice for diagnosis of abdominal pain, MRI is a very useful method to examine gynecological organs in older as well as young women. In addition, the continuous observation of PID with imaging studies is important to understand the clinical course.

On the other hand, type 2 diabetes mellitus (T2DM) is a disease with a lot of complications such as hyperglycemia, inflammation, and immune disorders. Therefore, patients with T2DM have an increased susceptibility to infection such as asymptomatic bacteria, urinary tract infections, and non-sexually transmitted genital infections, especially when glycemic control is poor [4, 5]. In addition, under hyperglycemic conditions, we have to be aware of the possibility of rare infection, which is seldom observed in healthy conditions.

Here we report a rare case of large pyosalpinx in an elderly patient with well-controlled T2DM. First, it is very rare that pyosalpinx is observed in older women. Second, this case report shows that various rare inflammatory diseases could be induced even when good glycemic control is obtained, suggesting that we should always pay attention to such rare inflammatory diseases in all patients with T2DM.

## Case presentation

**Fig. 1:**
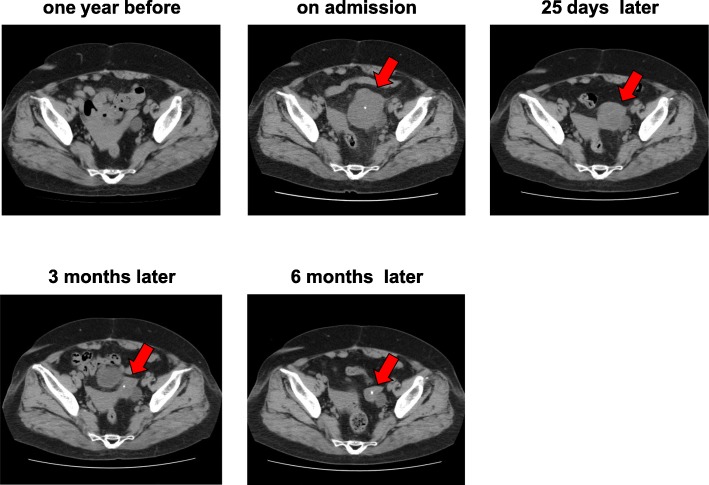
Abdominal computed tomography (CT) one year before (*upper left panel*), on admission (*upper middle panel*), 25 days later (*upper right panel*), 3 months later (*lower left panel*) and 6 months later (*lower middle panel*). Abnormal CT on admission showed a large tumor with calcification in the pelvis which was observed in abdominal CT one year before. Its size is as large as 65 mm in diameter (*upper middle panel*). Its tumor size was gradually reduced 25 days later (*upper right panel*), 3 months later (*lower left panel*) and 6 months later (*lower middle panel*) compared to that on admission (*upper middle panel*), but the tumor did not disappear completely even 6 months later. The arrows show the tumor which reduced in size at time course

**Fig. 2:**
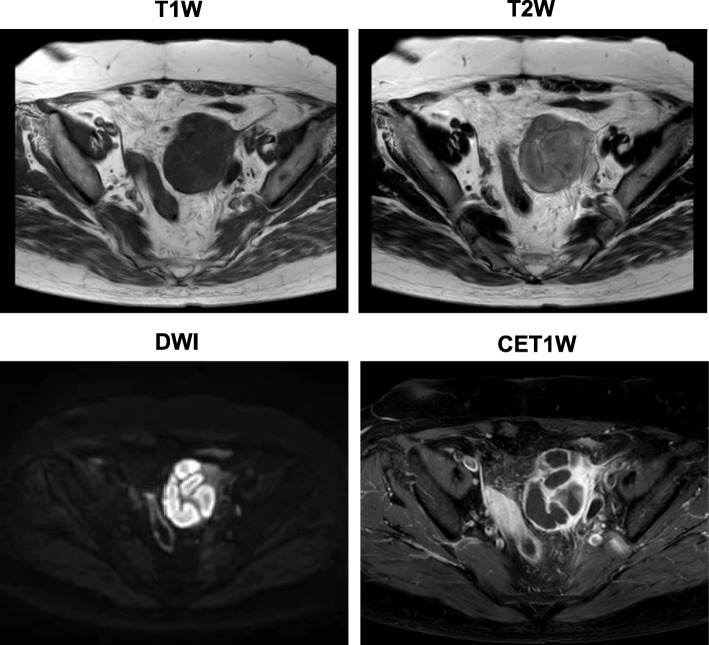
Magnetic resonance imaging axial T1-weighted images through the pelvis showed a markedly dilated fallopian tube posterior to the left ovary. Axial T2-weighted images showed a slightly higher intensity. Axial diffusion-weighted images and contrast-enhanced T1-weighted images showed a high intensity lesion at the same place. *CET1W* contrast-enhanced T1-weighted image, *DWI* diffusion-weighted image, *T1W* T1-weighted image, *T2W* T2-weighted image

A 72-year-old Japanese woman with a 10-year history of T2DM had symptoms of diarrhea and persistent pain in left lower abdomen for 2 days and visited the emergency room in Kawasaki Medical School. She had an approximately 10-year history of hypertension and dyslipidemia. At that time, she was taking 4 mg/day of benidipine hydrochloride and 20 mg/day of azilsartan for the treatment of hypertension, and 25 mg/day of alogliptin and 500 mg/day of metformin for T2DM, and 2.5 mg/day of rosuvastatin for dyslipidemia. She had no remarkable family history. She was a housewife and she did not smoke tobacco or drink alcohol. She had no past history of digestive disease or obstetrics and gynecology disease. She had mild tenderness to palpation in her abdomen. Her height and body weight were 150.0 cm and 69.5 kg, respectively. Her vital signs were as follows: blood pressure 150/87 mmHg, heart rate 110 beats/minute, and temperature 36.4 °C. Inflammation markers were markedly elevated: white blood cell (WBC), 20,110/μL (neutrophil, 89.0%); C-reactive protein (CRP), 16.12 mg/dL. Anemia and mild hypoalbuminemia were observed although their causes remained unknown: red blood cell, 304 × 10^4^/μL; hemoglobin (Hb), 9.3 g/dL; total protein (TP), 6.8 g/dL; and albumin (Alb), 3.2 g/dL. Her liver and renal function were within normal range as follows: aspartate aminotransferase (AST), 14 U/L; alanine aminotransferase (ALT), 9 U/L; gamma-glutamyl transpeptidase (γ-GTP), 8 U/L; lactate dehydrogenase (LDH), 202 U/L; creatinine (Cre), 0.81 mg/dL; blood urea nitrogen (BUN), 7 mg/dL; Na, 134 mEq/L; K, 3.8 mEq/L; Cl, 99 mEq/L; Cre clearance, 66.9 mL/minute; and urinary Alb, 15.1 mg/g·Cr. As shown in Fig. 1, her abdominal computed tomography (CT) on admission revealed a large tumor with calcification in left side of intrapelvis (upper middle panel) which was not observed in abdominal CT 1 year before (upper left panel). She had abdominal CT 1 year before by a urologist because bladder diverticulum was suspected at an annual medical checkup with abdominal ultrasonography. The tumor size was as large as 65 mm in diameter. In addition, as shown in Fig. 2, MRI showed a large tumor in left side of intrapelvis at the same lesion site observed in CT. An axial T1-weighted (T1W) image through the pelvis showed a markedly dilated fallopian tube posterior to the left ovary (upper left panel). Axial T2-weighted (T2W) image showed a slightly higher intensity (upper right panel). Axial diffusion-weighted (DW) image and contrast-enhanced T1W image showed a high intensity lesion at the same place (lower left and right panels). Based on these findings, we finally diagnosed her as having pyosalpinx.

**Fig. 3:**
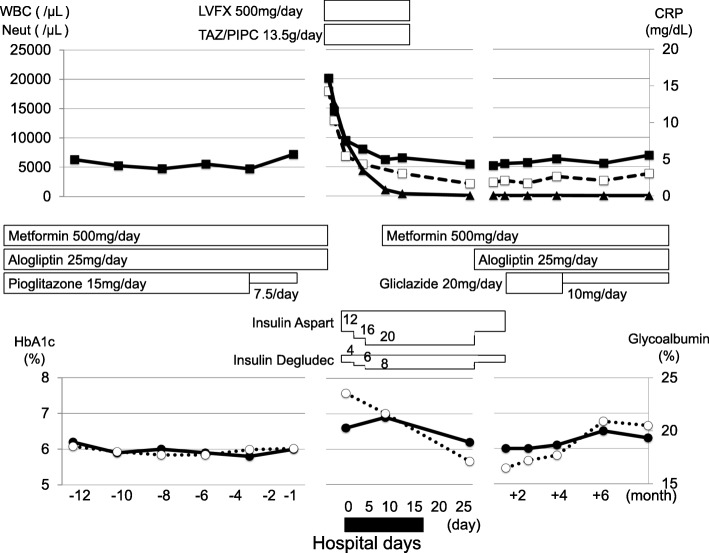
Time course of medication and inflammation markers. *Closed triangle*, CRP; *closed square*, WBC; *open square*, Neut; *closed circle*, HbA1c; *open circle*, glycoalbumin. *CRP* C-reactive protein, HbA1c, *LVFX* levofloxacin, *Neut* neutrophil, *TAZ/PIPC* tazobactam/piperacillin, *WBC* white blood cell

On admission, she had symptoms of diarrhea and persistent pain in left lower abdomen, but there were no findings in physical and neurological examinations. Her glycemic control was relatively good: HbA1c, 6.6%; glycoalbumin, 23.6%. In addition, her HbA1c levels were around 6% for over 1 year with the medication (metformin 500 mg, alogliptin 25 mg). However, during the acute phase of infection, we treated her with intensive insulin therapy using insulin aspart and insulin glargine. Tumor makers were within normal range: carcinoembryonic antigen (CEA), < 1.0 ng/mL; cancer antigen (CA) 19-9, 6.0 U/mL; and CA-125, 11.0 U/mL. Pathogenic bacteria were not detected. Her *Treponema pallidum* hemagglutination (TPHA) was positive but rapid plasma reagin (RPR) for *Treponema pallidum* was negative. *Candida* antigen and β-D-glucan were negative. *Neisseria gonorrhoeae* deoxyribonucleic acid (DNA) and *Chlamydia trachomatis* DNA in the urine were negative. Although pathogenic bacteria were not detected, we started antibiotics therapy for pyosalpinx (13.5 g/day of tazobactam/piperacillin and 500 mg/day of levofloxacin) (Fig. 3). We discussed the necessity of surgery such as laparoscopy with gynecologists, but finally we selected antibiotics therapy without laparoscopy because her symptoms and laboratory data were very much improved. Her laboratory data 14 days after starting the antibiotics were improved and became within normal range (WBC, 5500/μL (neutrophil 47.4%); CRP, 0.04 mg/dl); we stopped antibiotics (Fig. 3). Laboratory data were within normal range even after stopping antibiotics. T2DM was well controlled with orally administered anti-diabetes drugs (metformin 500 mg, alogliptin 25 mg, and gliclazide 10 mg): HbA1c, 6.0%; glycoalbumin, 16.5%. The tumor gradually reduced in size: 25 days later (upper right panel, Fig. 1), 3 months later (lower left panel, Fig. 1), and 6 months later (lower middle panel, Fig. 1) compared to that on admission (upper middle panel, Fig. 1); however, the tumor did not disappear completely even 6 months later. Finally she was discharged without any symptoms and/or problems. After discharge, she had no symptoms and/or problems, and her inflammation markers remained within normal levels for at least 6 months.

## Discussion

In this case report, we show a case of pyosalpinx in an elderly patient with well-controlled T2DM. Since her glycemic control was relatively good for over 1 year, it seemed that she was not in an immunocompromised state. However, she had a rare PID pyosalpinx while under good glycemic control. In addition, although laboratory data were within normal range even after stopping antibiotics, the tumor did not disappear completely even after 6 months. Therefore, we should think that patients with T2DM are immunocompromised hosts even when good glycemic control is obtained, and we should always pay attention to such rare inflammatory diseases in all patients with T2DM.

Pyosalpinx is a rare gynecological infection, especially in elderly women [1]. In addition, it is thought that infectious disease is not easily induced in patients with T2DM under good glycemic control. In this case report, however, we showed a case of large pyosalpinx in an elderly patient with well-controlled T2DM. To the best of our knowledge, this is a very rare case of PID especially in elderly patients with T2DM with good glycemic control.

The annual incidence of PID in women from 15 to 39 years of age is 10 to 13 per 1000 women, with a peak incidence of approximately 20 per 1000 women at 20 to 24 years. In addition, pyosalpinx accounts for approximately 16% of all cases of PID and it is most commonly observed in women aged from 20 to 40 [1]. Therefore, pyosalpinx is regarded as a young woman’s disease and it is rare that it is observed in older women.

On the other hand, it was reported that the presence of diabetes was associated with an increase of infectious disease [4, 5]. In fact, the presence of diabetes increases absolute risk, including urinary tract infection, genital infections, and PID by approximately 8%, although its precise mechanism remains unknown. In addition, high glucose concentration in urine increases growth of bacteria which also leads to an increase of infection in patients with T2DM. Therefore, in general, older women with T2DM and/or hyperglycemia more easily succumb to various rare infectious diseases compared to older women who do not have diabetes, although pyosalpinx is rare in older women.

Regarding PID, there is no single imaging or laboratory data with which we can correctly perform a definite diagnosis. MRI is a very useful method to examine and to diagnose gynecological organs in elderly as well as young women. DW images are particularly helpful for the diagnosis of PDI. MRI images in the pelvis show a markedly dilated fallopian tube posterior to the ovary, edema surrounding the fallopian tube, and thickened and enhanced tube wall with active inflammation [3]. However, it is rare for physicians to select MRI as a first choice for diagnosis of abdominal pain. In this case, we performed MRI after CT examination. Once we diagnosed this patient as having pyosalpinx, we followed up with CT.

As a therapy for PID, it is recommended that antibiotics therapy is started as soon as possible for patients with a high risk factor [6]. Therefore, although pathogenic bacteria were not detected, we started antibiotics therapy. In addition, although inflammation markers in this patient were promptly decreased with antibiotics and became within normal range (Fig. 3), the size of the pyosalpinx was not easily decreased. As shown in Fig. 1, pyosalpinx size decreased compared to that on admission but the tumor did not disappear. These findings suggest that we should know that pyosalpinx does not necessarily disappear within a short period of time even when inflammation markers are normalized and that we should continue to follow up the tumor for a long enough period by performing image inspection such as CT or MRI.

## Conclusions

In conclusion, we should keep in mind that elderly patients with T2DM are immunocompromised hosts and thereby they could have rare PIDs such as pyosalpinx even when good glycemic control is obtained for a long period of time.
